# Adrenocortical oncocytic neoplasm presenting with Cushing's syndrome: a case report

**DOI:** 10.1186/1752-1947-2-228

**Published:** 2008-07-13

**Authors:** Ozlem Yersal Kabayegit, Dilek Soysal, Gonca Oruk, Bahar Ustaoglu, Umut Kosan, Serife Solmaz, Arzu Avci

**Affiliations:** 1 Department of 1st Internal Medicine, Izmir Ataturk Training Hospital, Izmir, Turkey; 2 Department of Endocrinology, Izmir Ataturk Training Hospital, Izmir, Turkey; 3 Department of Pathology, Izmir Ataturk Training Hospital, Izmir, Turkey

## Abstract

**Introduction:**

Oncocytic neoplasms occur in several organs and are most commonly found in the thyroid, kidneys and salivary glands. Oncocytic neoplasms of the adrenal cortex are extremely rare and are usually non-functioning.

**Case presentation:**

We report the case of an adrenocortical oncocytic neoplasm with uncertain malignant potential in a 31-year-old man with Cushing's syndrome. The patient had been operated on following diagnosis of a 7 cm adrenal mass. Following surgery, the Cushing's syndrome resolved. The patient is still alive with no metastases one year after the surgery.

**Conclusion:**

Adrenocortical oncocytic neoplasms must be considered in the differential diagnosis of both functioning and non-functioning adrenal masses.

## Introduction

Adrenocortical neoplasms are the most frequent abnormalities of the adrenal cortex, but only a small fraction of patients also present with endocrine disorders [1]. Most of these lesions are clinically silent and are detected incidentally. The prevalence of adrenal tumors in the general population is around 1%, increasing with age to 6% in the elderly.

Oncocytic neoplasms are composed of oncocytic tumor cells, which are characterized by having large, eosinophilic, granular cytoplasm owing to the aberrant accumulation of mitochondria [2]. The most commonly reported sites for oncocytic neoplasms are the thyroid, kidneys and salivary glands. Oncocytic neoplasms of the adrenal cortex are extremely rare. Most adrenocortical oncocytic neoplasms are benign and non-functioning and are detected incidentally. We report the case of a patient with a functioning adrenal oncocytic neoplasm who presented with Cushing's syndrome.

## Case presentation

**Figure 1:**
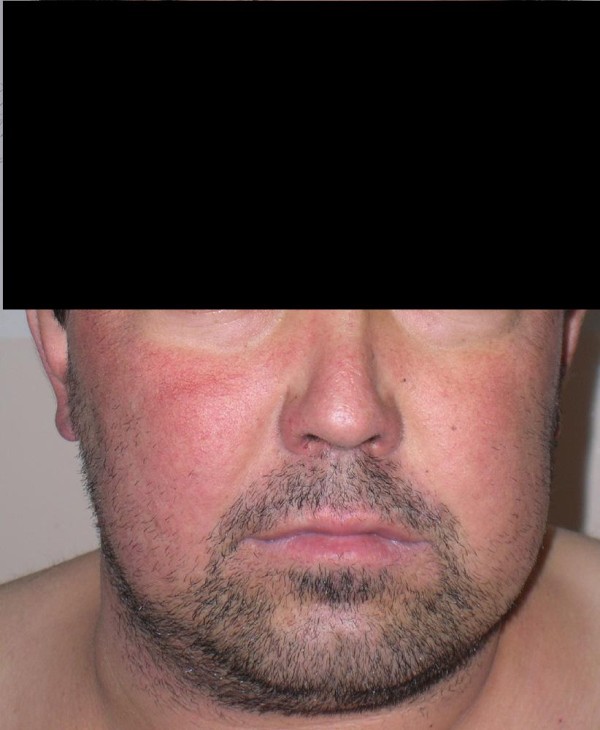
Plethoric moon face of the patient.

**Figure 2:**
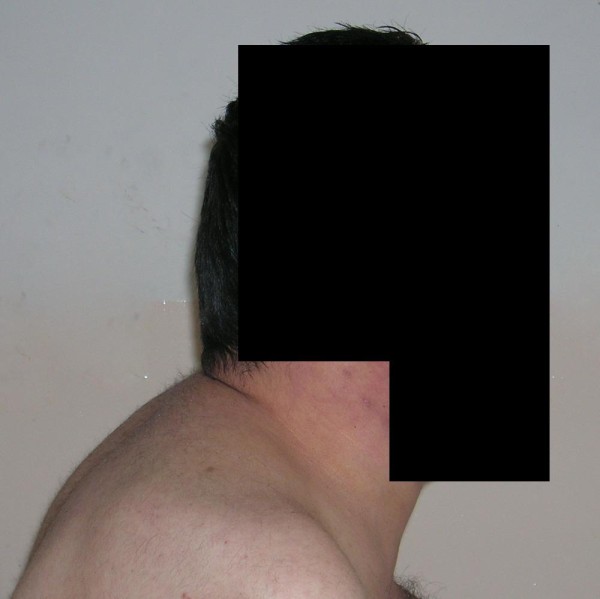
Truncal obesity of the patient.

**Figure 3:**
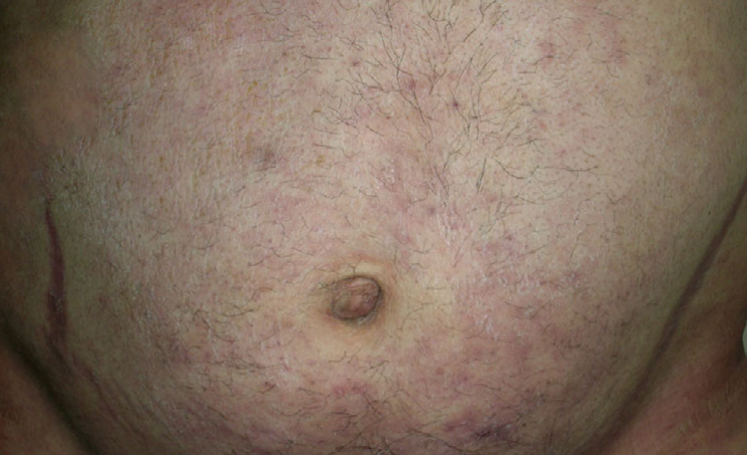
Abdominal cutaneous striae of the patient.

A 31-year-old man with progressive weight gain and fatigue for 10 years was admitted to hospital. He had developed edema of his hands and face and back pain during the previous two years. Physical examination revealed a typical cushingoid appearance including a plethoric moon face, truncal and centripetal obesity and abdominal cutaneous striae (Figure 1, Figure 2, Figure 3). His blood pressure on admission was 160/90 mmHg.

Laboratory investigations revealed leukocytosis and normal serum electrolytes and normal fasting and post-prandial glucose levels. Cushing's syndrome was suspected, and further endocrinological tests were performed to determine the etiology. Diurnal variation of plasma cortisol was not observed. The plasma cortisol level was not suppressed by 1 mg overnight or by 8 mg of standard dexamethasone. Plasma adrenocorticotropic hormone (ACTH) level was markedly suppressed (less than 10 pg/ml). Plasma free T_3_, free T_4_, thyroid-stimulating hormone (TSH), follicle-stimulating hormone (FSH), luteinizing hormone (LH), and urinary catecholamine levels were all within normal limits. There were no increases in the plasma concentrations of parathormone, calcitonin, testosterone, dehydroepiandrosterone sulphate, androstenedione or 24-hour urinary calcium excretion (Table 1).

**Table 1:** Laboratory evaluation

	Patient	Normal range
White blood cell (K/μl)	17.3	4 to 10
Calcium (mg/dl)	9.7	9 to 12
Carcinoembryonic antigen (ng/ml)	3.61	
24-hour urinary free cortisol (μg/24 hour)	350	
Plasma adrenocorticotropic hormone (pg/ml)	< 10	7.4 to 55.7
Plasma basal cortisol (μg/dl)	41.45	
Cortisol (after 1 mg dexamethasone) (μg/dl)	37.97	
Cortisol (after 2 mg dexamethasone) (μg/dl)	13.40	
Cortisol (after 8 mg dexamethasone) (μg/dl)	35.08	
Free T_3 _(pg/ml)	4.65	2.5 to 4.9
Free T_4 _(ng/dl)	0.969	0.8 to 1.9
Thyroid-stimulating hormone (μIU/ml)	1.63	0.4 to 5

Chest X-ray was normal. Dual-energy X-ray absorptiometry (DEXA) scanning demonstrated osteopenia at the femur head (T score, -2.46). T scores of the trochanter, Ward's triangle, L3, and L4 were -2.54, -4.07, -4.42 and -3.36, respectively, compatible with osteoporosis. Computed tomography of the abdomen revealed a 7 cm × 5 cm mass in the right adrenal gland. The patient underwent surgery for a right adrenalectomy. The pathologic diagnosis was adrenal oncocytic neoplasm with uncertain malignant potential according to the criteria proposed by Bisceglia et al. [3]. The Weiss score of the tumor was 2 (diffuse architecture and capsular invasion), compatible with a benign neoplasm.

**Figure 4:**
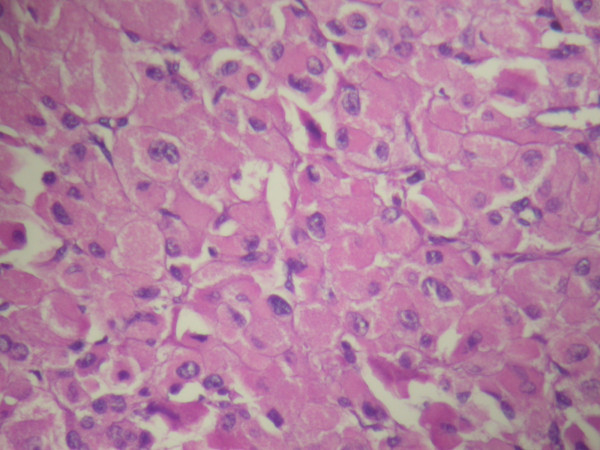
**Neoplasm composed of diffuse oncocytic cells**. The tumor was composed of polygonal oncocytes with granular, eosinophilic cytoplasm. Hematoxylin and eosin, magnification ×200.

**Figure 5:**
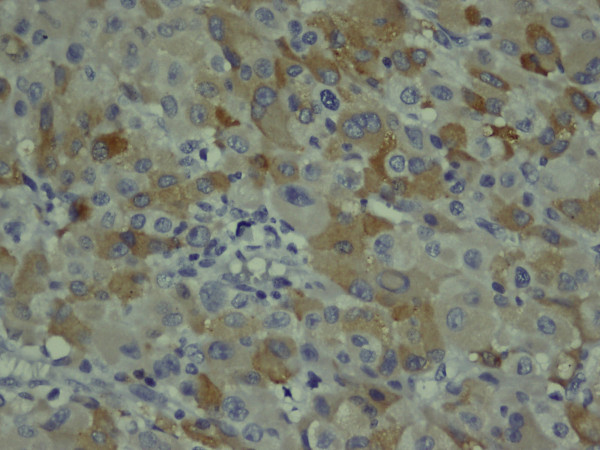
**The tumor cell cytoplasms showing focally positive immunoreactivity for inhibin immunostaining**. Immunohistochemical stain, magnification ×400.

**Figure 6:**
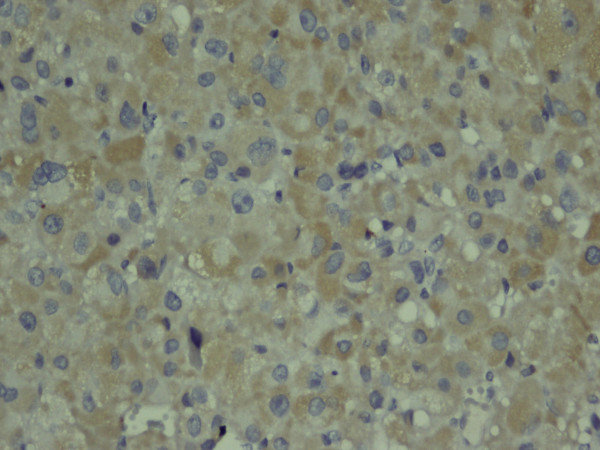
**The tumor cell cytoplasms showing focally positive pancytokeratin immunostaining**. Immunohistochemical stain, magnification ×400.

**Figure 7:**
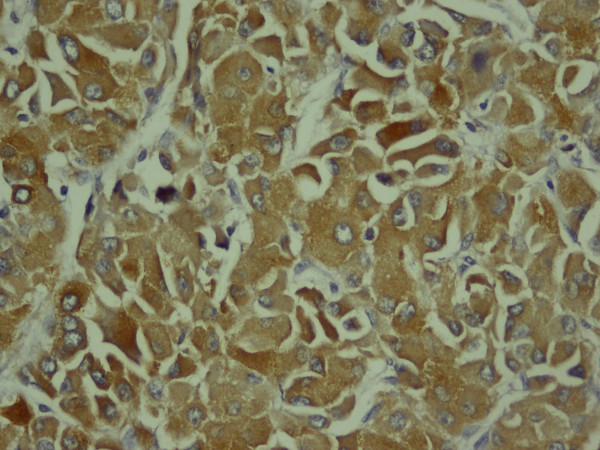
**The tumor cell cytoplasms showing focally positive synaptophysin immunostaining**. Immunohistochemical stain, magnification ×400.

The tumor measured 7 cm at its greatest dimension, weighed 105 g and was surrounded by an intact capsule. The tumor was composed of polygonal oncocytes with granular, eosinophilic cytoplasm (Figure 4). The tumor cells were arranged in a diffuse pattern. The mitotic rate was 1 per 50 high-power fields. There were no atypical mitotic figures and no necrosis. Capsular invasion was identified in one focus. Inhibin and pancytokeratin were focally positive (Figure 5, Figure 6). The tumor was immunoreactive for synaptophysin and negative for p53 (Figure 7).

From these findings, the final diagnosis for this patient with Cushing's syndrome was of a functioning adrenocortical oncocytic neoplasm.

The Cushing's syndrome improved gradually after the surgery. The patient is still alive with no metastases and is receiving glucocorticoid replacement therapy 1 year after the surgery. His blood pressure and electrolyte levels remain normal.

## Discussion

Oncocytic tumors originating from the adrenal cortex are extremely rare. To date, only 46 cases have been reported, and include 24 oncocytomas, 6 oncocytic neoplasms of uncertain malignant potential and 16 oncocytic carcinomas. The patients had a wide age range (27 to 74 years) with a significant female to male predominance. The neoplasms varied in size from 2.2 cm to 15 cm and in weight from 22 g to 865 g. All arose in the adrenal gland with the exception of two cases, which occurred in heterotopic retroperitoneal adrenal tissue [4,5]. Patients were usually not initially referred to an oncologist. In all but one case, oncocytic neoplasms were diagnosed incidentally or during investigation for symptoms that were not attributable to the tumor, such as abdominal pain, hematuria, essential hypertension, episodic vomiting, ascites and edema of the lower extremities [6,7]. El-Naggar et al. [8] reported the case of one patient who suffered from abdominal pain caused by tumor infiltration to the adjacent vena cava and liver.

Oncocytic neoplasms of the adrenal cortex were non-functioning with the exception of two cases. Xiao et al. [9] identified an adrenocortical oncocytoma in a 53-year-old woman who presented with Cushing's syndrome. Erlandson and Reuter [10] reported a female patient who was incidentally found to have a 5 cm virilizing adrenocortical oncocytoma and a coexisting urinary bladder tumor. After surgery, this patient's 17-ketosteroid level returned to normal.

Our patient had a functioning adrenocortical oncocytic neoplasm. He was symptomatic and admitted to the hospital because of suspected Cushing's syndrome. The laboratory findings were compatible with the syndrome, and further investigation revealed a mass in the right adrenal gland. After the surgery, the Cushing's syndrome resolved.

The biologic behavior of adrenocortical neoplasms is the most important practical problem. A combination of clinical, biochemical and histological features can differentiate benign and malignant adrenocortical tumors. Aside from the obviously malignant cases in which the diagnosis is based on common clinicopathologic features universally valid for tumors of any site, such as distant metastases, surgical unresectability and/or invasion of adjacent organs, the prediction of biologic behavior and clinical outcome can be difficult. There is no single histological parameter that is predictive of the clinical outcome and biologic behavior of adrenocortical oncocytic neoplasms. The Weiss system is the most widely used and accepted histological scheme to distinguish benign from malignant adrenal tumors [11]. According to this system, the presence of four or more of the nine criteria (high mitotic rate, atypical mitoses, high nuclear grade, low percentage of clear cells, necrosis, diffuse tumor architecture, capsular invasion, sinusoidal invasion and venous invasion) indicates a malignant neoplasm. However, the Weiss criteria have limitations. First, Weiss studied only 43 adrenocortical tumors of which 25 were benign and 18 were malignant tumors according to his classification. Second, patients with benign tumors in his series had a longer follow-up period than those with malignant tumors. Third, tumor tissue could be heterogeneous within the same lesion. Therefore, the Weiss score, even if established by experienced pathologists, cannot be completely reliable. Pohlink et al. [12] reported a patient with an adrenal incidentaloma, which was initially diagnosed as benign but on follow-up was reclassified as malignant because of local recurrence and pulmonary metastases.

Bisceglia et al. [3] proposed new criteria that modified the Weiss system. According to this system, if the tumor exhibits any of the major criteria (high mitotic activity, atypical mitoses or venous invasion), it is considered malignant; if the tumor exhibits any of the minor criteria (large size, necrosis, capsular or sinusoidal invasion), it is considered to have uncertain malignant potential; and none of these features indicates a benign tumor [3]. In this case, there were no atypical mitotic figures or venous invasion and the mitotic rate was 1 per 50 high-power fields, although capsular invasion was present in one focus. Therefore, this case was diagnosed as oncocytic neoplasm with uncertain malignant potential according to the system proposed by Bisceglia et al.

Adrenocortical oncocytomas are generally considered as benign neoplasms. In 22 of the 25 reported patients for whom follow-up information was available, no recurrence or metastases were observed within a follow-up period ranging from 1 to 99 months.

Borderline adrenal oncocytomas also seem to have a benign clinical behavior. Bisceglia et al. [3] reported four patients with a mean follow-up of 38.75 months (10 to 61 months) with no evidence of the disease. Lin et al. [7] reported two patients with uncertain malignant potential with a mean follow-up of 15.5 months (12 to 19 months) who had not experienced recurrence or metastases.

Recurrence and metastases have been described in patients with an adrenal oncocytic carcinoma. Kurek et al.[13] described a patient who exhibited widespread retroperitoneal infiltration 7 years after the removal of an adrenal tumor. Local invasion into the inferior vena cava and extension to the right atrium was observed in one case [6] and to the liver in another [8].

There was no evidence of metastases in our patient. The tumor was compatible with the diagnosis of an oncocytic neoplasm with uncertain malignant potential. The mass was surgically removed and no other therapy was given. The patient will be followed-up to check for any recurrence or metastases every 6 months.

## Conclusion

Although rare, adrenocortical oncocytic neoplasms must be considered among the differential diagnosis of both functional and non-functional adrenal masses. Clinical, biochemical and histological features must be evaluated together to assess the biologic behavior of these tumours.

## Abbreviations

ACTH: Adrenocorticotropic hormone; DEXA: Dual-energy X-ray absorptiometry; FSH: Follicle-stimulating hormone; LH: Luteinizing hormone; TSH: Thyroid-stimulating hormone.

## Competing interests

The authors declare that they have no competing interests.

## Consent

Written informed consent was obtained from the patient for publication of this case report and any accompanying images. A copy of the written consent is available for review by the Editor-in-Chief of this journal.

## Authors' contributions

OYK conceived of the study, made substantial contributions to the acquisition of data, drafted the initial manuscript and revised the draft over the course of submission. DS coordinated the design and drafting of the initial version and revised the draft over the course of submission. GO carried out endocrinological evaluation of the patient, made contributions to the acquisition of data and revised the draft wherever necessary. BU made contributions to the acquisition of data, participated in study design and coordination, and helped to draft the manuscript. UK made contributions to the acquisition of data, participated in study design and coordination, and helped to draft the manuscript. SS carried out suppression tests and helped to draft the manuscript. AA carried out pathologic assessment and revised the draft where necessary. All authors read and approved the final manuscript.
